# Supplementary material for: Identification of photoperiod-induced specific miRNAs in the adrenal glands of Sunite sheep (Ovis aries)
Source: Front Vet Sci. 2022 Jul 22;9:888207. doi: 10.3389/fvets.2022.888207 (PMC9354845; doi:10.3389/fvets.2022.888207)
Supplement: Supplementary file 1 [file Data_Sheet_1.ZIP › Supplementary file/miRDeep2 file.zip!/miRDeep2 file/Know_Structure/SPLP42A3/oar-mir-199a.pdf]

miRBase precursor : oar-mir-199a
Total read count : 48188
oar-miR-199a-3p read count : 28191
remaining reads : 19997

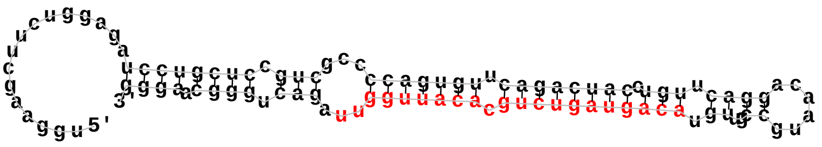

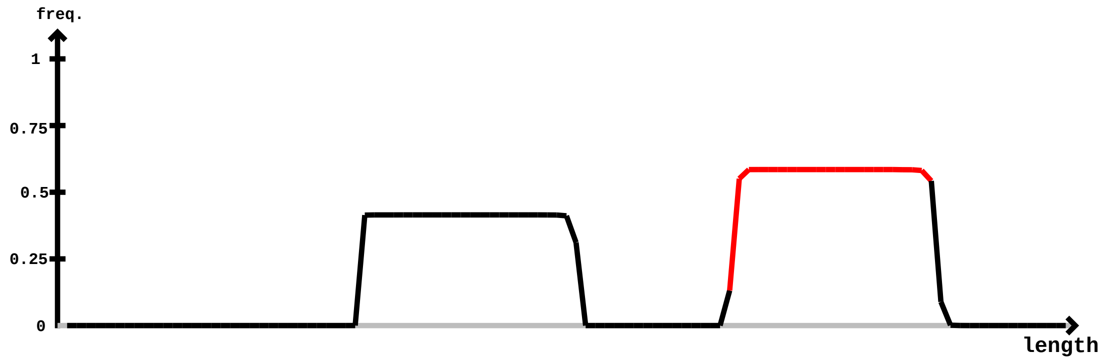

oar-miR-199a-3p

5'- uggaagcuucuggagauccugcuccgucgccccaguguucagacuaccuguucaggacaaugccguuguacaguagucugcacauugguuagacugggcaaggg -3' exp

.................(((((((.(((...(((((((.((((((((.(((.((((......))..)).))))))))))).)))))))...))).)))).))).	reads	mm	sample
.............................ccccaguguucagacuaccugu...	1	0	seq
.............................ccccaguguucagacuaccuguu..	3	0	seq
.............................ccccaguguucagacuaccuguuc...	3	0	seq
..............................cccaguguucagacuac...	1	0	seq
..............................cccaguguucagacuaccu...	6	0	seq
..............................cccaguguucagacuaccug..	14	0	seq
..............................cccaguguucagacuaccugu...	144	0	seq
..............................cccaguguucagacuaccuguu..	4798	0	seq
..............................cccaguguucagacuaccuguuc...	14989	0	seq
...............................ccaguguucagacuaccuguu..	8	0	seq
...............................ccaguguucagacuaccuguuc...	25	0	seq
................................caguguucagacuaccuguuc...	2	0	seq
.................................aguguucagacuaccuguu..	1	0	seq
..................................guguucagacuaccuguu..	1	0	seq
...................................uguucagacuaccuguu..	1	0	seq
....uacaguagucugcacauu..................	10	0	seq
....uacaguagucugcacauug.................	2	0	seq
....uacaguagucugcacauugg................	43	0	seq
....uacaguagucugcacauuggu...............	899	0	seq
....uacaguagucugcacauugguu..............	5114	0	seq
....uacaguagucugcacauugguua.............	264	0	seq
....uacaguagucugcacauugguuag............	5	0	seq
....acaguagucugcacauu..................	21	0	seq
....acaguagucugcacauug.................	5	0	seq
....acaguagucugcacauugg................	79	0	seq
....acaguagucugcacauuggu...............	865	0	seq
....acaguagucugcacauugguu..............	15573	0	seq
....acaguagucugcacauugguua.............	3605	0	seq
....acaguagucugcacauugguuag............	48	0	seq
....acaguagucugcacauugguuagG...........	1	1	seq
....acaguagucugcacauugguuaga...........	5	0	seq
....caguagucugcacauugg................	11	0	seq
....caguagucugcacauuggu...............	116	0	seq
....caguagucugcacauugguu..............	1158	0	seq
....caguagucugcacauugguua.............	350	0	seq

oar-miR-199a-3p

uggaagcuucuggagauccugcuccgucgccccaguguucagacuaccuguucaggacaaugccguuguacaguagucugcacauugguuagacugggcaaggg			
....caguagucugcacauugguuag............	7	0	seq
....caguagucugcacauugguuaga...........	2	0	seq
....aguagucugcacauuggu...............	2	0	seq
....aguagucugcacauugguu..............	5	0	seq
....aguagucugcacauugguua.............	1	0	seq
